# Long COVID’s Hidden Complexity: Machine Learning Reveals Why Personalized Care Remains Essential

**DOI:** 10.3390/jcm14113670

**Published:** 2025-05-23

**Authors:** Eleonora Fresi, Elisabetta Pagani, Federica Pezzetti, Cristina Montomoli, Cristina Monti, Monia Betti, Annalisa De Silvestri, Orlando Sagliocco, Valentina Zuccaro, Raffaele Bruno, Catherine Klersy

**Affiliations:** 1 Biostatistics & Clinical Trial Center, Fondazione IRCCS Policlinico San Matteo, 27100 Pavia, Italyc.klersy@smatteo.pv.it (C.K.); 2 Infectious Diseases Unit, Fondazione IRCCS Policlinico San Matteo, 27100 Pavia, Italy; 3 Operation Management Next Generation EU, ASST Cremona, 26100 Cremona, Italy; federica.pezzetti@asst-cremona.it; 4 Biostatistics and Clinical Epidemiology Unit, Department of Public Health, Experimental and Forensic Medicine, University of Pavia, 27100 Pavia, Italy; 5 Pneumology Unit, ASST Cremona, 26100 Cremona, Italy; 6 Intensive Care Unit Bolognini Hospital, ASST Bergamo Est, 24068 Seriate, Italy; orlando.sagliocco@asst-bergamoest.it; 7 Dipartimento di Scienze Cliniche Chirurgiche Diagnostiche e Pediatriche, Università di Pavia, 27100 Pavia, Italy

**Keywords:** PASC, cluster, phenotype, machine learning, severe COVID

## Abstract

**Background:** Long COVID can develop in individuals who have had COVID-19, regardless of the severity of their initial infection or the treatment they received. Several studies have examined the prevalence and manifestation of symptom phenotypes to comprehend the pathophysiological mechanisms associated with these symptoms. Numerous articles outlined specific approaches for multidisciplinary management and treatment of these patients, focusing primarily on those with mild acute illness. The various management models implemented focused on a patient-centered approach, where the specialists were positioned around the patient. On the other hand, the created pathways do not consider the possibility of symptom clusters when determining how to define diagnostic algorithms. **Methods:** This retrospective longitudinal study took place at the “Fondazione IRCCS Policlinico San Matteo”, Pavia, Italy (SMATTEO) and at the “Ospedale di Cremona”, ASST Cremona, Italy (CREMONA). Information was retrieved from the administrative data warehouse and from two dedicated registries. We included patients discharged with a diagnosis of severe COVID-19, systematically invited for a 3-month follow-up visit. Unsupervised machine learning was used to identify potential patient phenotypes. **Results:** Three hundred and eighty-two patients were included in these analyses. About one-third of patients were older than 65 years; a quarter were female; more than 80% of patients had multi-morbidities. Diagnoses related to the circulatory system were the most frequent, comprising 46% of cases, followed by endocrinopathies at 20%. PCA (principal component analysis) had no clustering tendency, which was comparable to the PCA plot of a random dataset. The unsupervised machine learning approach confirms these findings. Indeed, while dendrograms for the hierarchical clustering approach may visually indicate some clusters, this is not the case for the PAM method. Notably, most patients were concentrated in one cluster. **Conclusions:** The extreme heterogeneity of patients affected by post-acute sequelae of SARS-CoV-2 infection (PASC) has not allowed for the identification of specific symptom clusters with the most recent statistical techniques, thus preventing the generation of common diagnostic-therapeutic pathways.

## 1. Introduction

The British National Institute for Health and Care Excellence (NICE) describes long COVID or post-COVID syndrome (PCS) as the continuation of signs and symptoms that were present during or arose after a COVID-19 infection and persist for more than twelve weeks, with no other explanation for their persistence [1]. In contrast, the United States National Institutes of Health (NIH) refers to Long COVID as sequelae that persist beyond four weeks from the onset of the initial infection, as the definition provided by the Centers for Disease Control and Prevention (CDC) [2]. Nowadays, post-acute sequelae of SARS-CoV-2 infection (PASC) are defined as symptoms that persist, relapse, or arise 30 or more days after a SARS-CoV-2 infection [3].

Many studies examined the residual symptoms reported after contracting SARS-CoV-2, including the incidence, risk factors, treatment, and management of long COVID [4,5]. Considering all these factors, it is evident that this virus can potentially result in lasting health consequences [6]. Long COVID can impact individuals who experienced mild symptoms during their initial illness, as well as those who battled against more severe forms of the infection [7,8]. Long COVID can develop in any individual who has had COVID-19, regardless of the severity of the initial infection or the treatment they received. This includes patients treated in hospital wards or intensive care units, requiring oxygen therapy, continuous positive airway pressure, or invasive ventilation, and those not hospitalized [9].

Several studies have used various methodologies to identify long COVID phenotypes, including hierarchical cluster analysis, latent class analysis, and phenotype semantic similarity methods. However, these approaches have significant limitations. First, many studies have combined patients with varying acute disease severity, obscuring potential differences in post-acute patterns between these groups. Inconsistencies between previous studies are evident in the different numbers and types of clusters identified across the literature. For instance, some studies describe three long COVID phenotypes based primarily on symptom severity, while others identify up to six distinct clusters characterized by different pre-existing comorbidities.

Severity-based analysis is essential for several reasons. First, patients with severe COVID-19 might have distinct pathophysiological mechanisms underlying their post-acute symptoms, including more extensive organ damage, more intense systemic inflammation, and complications related to prolonged hospitalization. Second, this population represents a significant care burden and requires dedicated management strategies.

Given the known association between COVID-19 and long-term cardiovascular alterations, it is particularly important to investigate potential phenotypic patterns linked to cardiovascular manifestations. Cardiovascular complications represent one of the most concerning manifestations of PASC, with growing evidence suggesting specific pathophysiological mechanisms such as persistent vascular inflammation, alterations in the renin–angiotensin–aldosterone system, and direct endothelial damage.

Several studies have used various methodologies to identify long COVID phenotypes, including hierarchical cluster analysis, latent class analysis, and phenotype semantic similarity methods [10,11,12,13]. However, these approaches have significant limitations. First, many studies have combined patients with varying acute disease severity, obscuring potential differences in post-acute patterns between these groups [12,13]. Inconsistencies between previous studies are evident in the different numbers and types of clusters identified across the literature. For instance, some studies describe three long COVID phenotypes based primarily on symptom severity [10,11], while others identify up to six distinct clusters characterized by different pre-existing comorbidities [14,15]. Severity-based analysis is essential for several reasons. First, patients with severe COVID-19 might have distinct pathophysiological mechanisms underlying their post-acute symptoms, including more extensive organ damage, more intense systemic inflammation, and complications related to prolonged hospitalization [12,13]. Second, this population represents a significant care burden and requires dedicated management strategies. Given the known association between COVID-19 and long-term cardiovascular alterations, it is particularly important to investigate potential phenotypic patterns linked to cardiovascular manifestations. Cardiovascular complications represent one of the most concerning manifestations of PASC, with growing evidence suggesting specific pathophysiological mechanisms such as persistent vascular inflammation, alterations in the renin–angiotensin–aldosterone system [16,17], and direct endothelial damage [13,14,15,18].

Several studies examined the prevalence and manifestation of different symptom phenotypes to comprehend the underlying pathophysiological mechanisms associated with these symptoms [19]. However, these studies did not differentiate between patients based on the severity of their acute illness [20,21]. They identified various phenotypes in diverse populations of COVID patients, including both those who were hospitalized and those who were not [22]. These studies are focused on deciphering the pathophysiological mechanisms underlying PASC.

Numerous articles outlined specific approaches for the multidisciplinary management and treatment of these patients, focusing primarily on those with mild acute illness [23]. The various management models implemented focused on a patient-centered approach, where the specialists involved were positioned around the patient [24]. The created pathways did not consider the possibility of symptom clusters when determining how to define diagnostic algorithms.

Thus, due to the complexity of the issue, a comprehensive and universally accepted definition is challenging.

Our research seeks to determine how common lingering symptoms are, three months after patients have been released from the hospital following a severe case of COVID-19. Lombardy was a region with a high rate of COVID-19 infections during the initial phases of the pandemic. Our primary research question is whether distinct symptom clusters can be identified among these patients, potentially reflecting different phenotypes of PASC. Identifying such clusters may support the development of targeted follow-up strategies, improve care coordination, and inform standardized treatment pathways for long COVID patients.

## 2. Materials and Methods

### 2.1. Study Design

This is a retrospective longitudinal study part of a larger research project funded by Fondazione CARIPLO, the “Chronic diseases management after the COVID-19 epidemic trigger. Capturing data, generating evidence, suggesting actions for health protection. The CHANCE Project” (cod. 2020-4238). This sub-project took place at the “Fondazione IRCCS Policlinico San Matteo”, Pavia, Italy (SMATTEO) and at the “Ospedale di Cremona”, ASST Cremona, Italy (CREMONA); the study was approved by the ethical committee of Pavia (26 July 2022, protocol number 0036061/22) as well as by the ethical committee of Val Padana (30 September 2022, protocol number 34131).

### 2.2. Data Source

Discharge data on hospitalization were retrieved from the administrative databases of both hospitals, and follow-up data were derived from dedicated clinical COVID registries maintained at both hospitals. Patients with multimorbidities were identified through the ICD9-CM discharge diagnoses up to the 6th.

### 2.3. Study Population

Individuals with residual symptoms correlated with PASC were enrolled during the outpatient follow-up visit at 3 months after discharge from the two medical facilities. Subjects discharged between March 2020 and March 2022 with a diagnosis of severe COVID-19 were eligible for the study. Specifically, subjects who required either CPAP (Continuous Positive Airway Pressure) or Endotracheal Intubation and exhibited residual symptoms at the 3- to 4-month visit were included. The following discharge ICD9-CM diagnoses were considered: codes 48041, 51891, 9604, 311, 9670, 9671, 9672, and 9390. Table 1 reports the descriptive characteristics of the study population, including sex, age, and comorbidities (which were grouped according to ICD9-CM chapter), stratified by center.

During the outpatient visit, information about the presence of residual symptoms was collected, listed in Supplementary Table S1.

### 2.4. Data Analysis

All analyses were performed using the R (v. 4.3.1) software [25]. We used the Fisher exact test to compare patient characteristics between the two hospitals. The prevalence of each category of symptoms, collected at the 3-month follow-up visit, was computed together with its exact binomial 95% confidence interval (95% CI). A list of the 36 variables included in the analysis is reported in Supplementary Table S1. It is important to note that all symptoms were analyzed as individual binary variables, without any aggregation, to preserve the specificity and granularity of each clinical feature.

In order to elicit potential different aggregations of patients, we plotted the entire case series over the first two components of a principal component analysis (PCA). For comparison, PCA was also run on the random dataset. Additionally, other non-linear dimensionality reduction techniques were investigated, such as t-distributed Stochastic Neighbor Embedding (t-SNE) and Uniform Manifold Approximation and Projection (UMAP).

More formally, we applied a series of unsupervised machine learning techniques, such as hierarchical clustering (either agglomerative or divisive), partition around medoids (PAM), k-means, and Density-Based Spatial Clustering of Applications with Noise (DBSCAN). These techniques attempt to find subgroups of patients that share common characteristics and differ from the other subgroups. To rank the performance of such methods, we calculated the following indices that measure the separation between potential clusters (the higher the better): the average silhouette width, with a value >0.5 generally considered as an acceptable performance, the separation index (range 0–1), and the cophenetic correlation coefficient (range |0–1|). To further discriminate between these methods, we computed the entropy, where lower values indicated lower heterogeneity within clusters. The results of the clustering processes were reported graphically as dendrograms or cluster plots. Patients for whom more than 50% of the selected variables were missing did not enter the machine learning approach. A detailed description of these analyses is reported in the Supplementary Materials.

Parameter sensitivity analyses were conducted for clustering methods that require parameter specification (e.g., number of clusters for k-means and PAM, eps value for DBSCAN) to ensure robust results. Age and sex were included as variables in the clustering analysis to account for their potential influence on symptom patterns. Additionally, we performed exploratory stratified clustering analyses by age group (≥65 vs. <65 years) to assess whether age-specific symptom patterns might emerge more clearly.

To assess whether the exclusion of patients with more than 50% missing data introduced any systematic bias, we compared key demographic and clinical variables between included and excluded patients (Supplementary Table S3).

## 3. Results

### 3.1. Patient

In our study, we included 382 patients discharged with a diagnosis of severe COVID-19 and a 3-month follow-up visit. Their demographic and clinical characteristics are shown in Table 1: about one-third of patients were older than 65 years, and a quarter were female; 25% of this case series had had endotracheal intubation during their hospitalization; more than 80% of patients had multimorbidities. Diagnoses related to the circulatory system were notably the most frequent, including 46% of cases, followed by those within the endocrine ICD9-CM chapter at 20%. All other diagnoses had a prevalence below 10%. Notably, 40% and 30% of diagnoses were unspecified and symptomatic, respectively. Table 2 reports the prevalence of symptoms at follow-up. About 70% of patients (*N* = 253) attending the outpatient clinic had residual symptoms at the 3-month follow-up visit, with 40% of them with 2 or more symptoms. Dyspnea prevalence was largely the highest, with 60% of patients affected. Fatigue (40%) and neuropsychological symptoms (30%) were other frequent symptoms.

### 3.2. Unsupervised Machine Learning for the Identification of Patient Aggregation

Out of 253 subjects with residual symptoms, 19 patients (7.5%) with more than 50% of missing data were excluded from the analysis. Therefore, these analyses included 234 patients with symptoms and sufficient available data.

**Figure 1:**
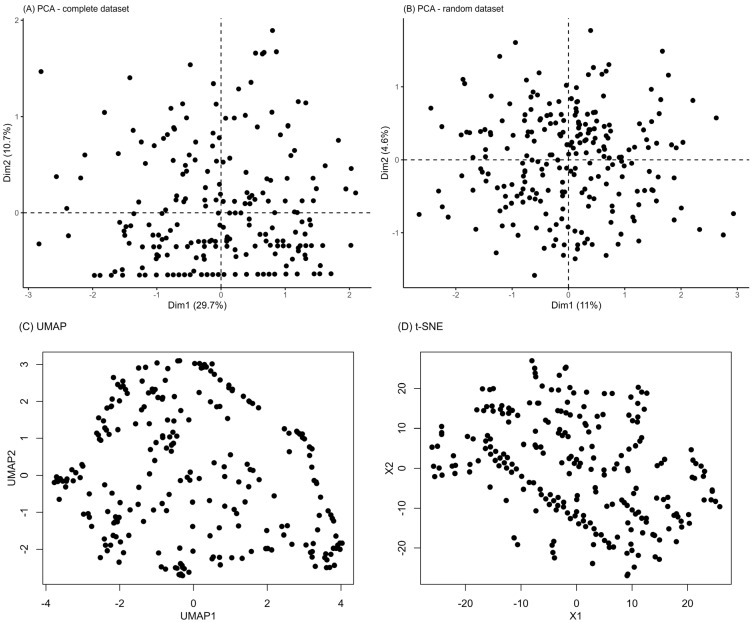
Dimensionality reduction techniques applied to the dataset. PCA plot of the first two principal components for patient data (**A**) and a randomly generated dataset (**B**). Uniform Manifold Approximation and Projection (UMAP) (**C**) and t-distributed Stochastic Neighbor Embedding (t-SNE) (**D**).

As shown in Figure 1A, there was no clustering tendency at PCA, comparable to the PCA plot of a random dataset (Figure 1B). Similarly, neither UMAP (Figure 1C) nor t-SNE (Figure 1D) revealed distinct or stable clusters in the symptom data.

**Figure 2:**
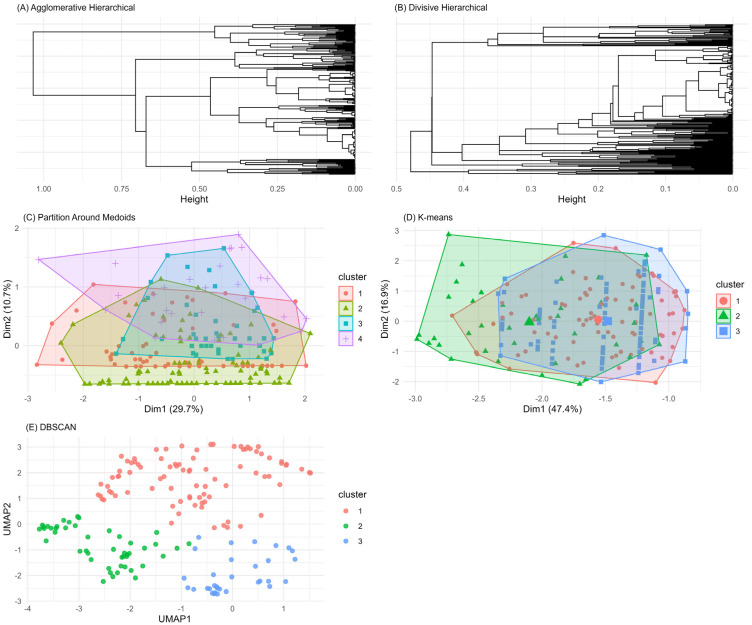
Clustering results for the patient dataset using five different algorithms: (**A**) agglomerative hierarchical clustering, (**B**) divisive hierarchical clustering, (**C**) PAM, (**D**) k-means, and (**E**) DBSCAN. Each panel illustrates the clustering structure detected by the corresponding method.

The unsupervised machine learning approach confirms these findings. Indeed, while dendrograms for the hierarchical clustering approach may visually indicate some clusters (Figure 2A,B), this is not the case for the PAM method (Figure 2C) and k-means (Figure 2D). Notably, for the PAM method, most patients were concentrated in one cluster (181 out of 234). All these methods were applied combined with parameter sensitivity analyses (e.g., number of clusters for k-means and PAM, eps value for DBSCAN). Moreover, the internal validation indices did not support the validity of patient aggregation, as evidenced by inferior values (Table 3). Specifically, the average silhouette values were below the acceptable threshold of 0.5 across all cases, indicating that the clusters might overlap or were not well-defined. Similarly, the separation index was close to 0, confirming the lack of separation between the hypothetical clusters. Among the clustering methods tested, DBSCAN (Figure 2E) showed the best performance based on a higher silhouette index (0.47) and separation index (0.55). However, further investigation of the clinical characteristics of patients within the clusters identified by DBSCAN did not reveal any clinically meaningful distinctions (Supplementary Table S3).

The stratified clustering analyses by age group (≥65, 96 patients vs. <65, 138 patients) did not yield well-defined or clinically meaningful clusters either, consistent with the results from the overall cohort, further supporting the conclusion that the absence of clustering reflects true heterogeneity in the presentation of long COVID symptoms rather than a methodological limitation.

## 4. Discussion

Patients included in this study were recruited from two major Centers in Lombardy (IRCCS Policlinico San Matteo Foundation and Cremona Hospital), areas with a high incidence of COVID-19 during the first two waves of the pandemic. The study aimed to evaluate the prevalence of post-acute sequelae of SARS-CoV-2 infection (PASC) symptoms in patients discharged after a severe COVID-19. It sought to identify symptom-based patient clusters to facilitate structured management pathways. Utilizing a retrospective longitudinal design within a larger project funded by Fondazione CARIPLO, the researchers analyzed hospital discharge and follow-up data from COVID registries. A total of 382 patients with severe COVID-19 were included. At the 3-month follow-up, 70% of patients exhibited residual symptoms, predominantly dyspnea, fatigue, and neuropsychological issues.

The results of our investigation, in particular the application of an unsupervised machine learning approach, indicate that there was no discernible clustering of patients, thus precluding the identification of specific phenotypes among individuals, systematically assessed three months after discharge with a diagnosis of severe COVID-19 and residual symptoms of PACS.

Additionally, we observed a limited number of patients who required continuous positive airway pressure (CPAP) or endotracheal intubation during their hospitalization. This observation can be attributed to the fact that patients requiring such interventions were less likely to be discharged alive and, consequently, were unable to participate in the three-month follow-up visit.

Our analysis revealed that the study population was characterized by a remarkably high prevalence of multimorbidities (84.4%), with circulatory and endocrine diseases being the most commonly observed comorbid conditions. This highlights the complexity of managing post-COVID-19 patients, especially those with pre-existing health conditions, and underscores the importance of comprehensive and tailored medical care to address their diverse needs.

Regarding cardiovascular manifestations specifically, our analysis showed that cardiovascular symptoms were present in 17.2% of patients at the 3-month follow-up, with a slightly higher prevalence in patients from San Matteo Hospital (20.3%) compared to those from Cremona Hospital (14.1%). However, the clustering analysis did not highlight patient groupings primarily characterized by cardiovascular manifestations. This observation suggests that cardiovascular symptoms, while clinically relevant, tend to present in variable combinations with other post-COVID symptoms rather than constituting a distinct and isolated phenotype.

The most common symptoms reported in clusters of PACS patients vary but generally include a range of physical, cognitive, and psychiatric manifestations. Fatigue emerges as a predominant symptom across multiple studies, often accompanied by dyspnea (shortness of breath) and cognitive impairments such as forgetfulness and memory impairment. For instance, one study identified clusters including fatigue alone and combinations of fatigue with other symptoms like dyspnea, chest pain, and cognitive disturbances [26]. Similarly, another study highlighted fatigue, dyspnea, and myalgia as the most common symptoms, with women reporting more symptoms than men [27]. Psychiatric symptoms, including anxiety and depression, are also frequently reported among long COVID patients. A systematic review found sleep disturbances, depression, post-traumatic stress symptoms, anxiety, and cognitive impairments to be common psychiatric manifestations [28]. Moreover, the risk factors for developing psychiatric symptoms include being female and having a previous psychiatric diagnosis [29]. The heterogeneity of PASC symptoms is further evidenced by the identification of symptom clusters such as gastrointestinal, musculoskeletal, neurocognitive, and cardiopulmonary in one study, with neurocognitive symptoms being associated with increased odds of depression and anxiety [30]. Another study proposed three phenotypes of PASC based on symptom severity, with the severe phenotype characterized by fatigue, cognitive impairment, and depression [20]. Research also indicates that the symptomatology of PASC can evolve over time, with variations in symptom clusters observed across different waves of the pandemic and about SARS-CoV-2 variants [31]. Additionally, the presence of symptoms like joint pain, chest discomfort, and hair loss points to the multisystemic nature of PASC [32,33]. In summary, long COVID presents with a wide array of symptoms, predominantly fatigue, dyspnea, cognitive impairment, and psychiatric symptoms, with significant variability in symptom clusters among patients [34]. To highlight the natural history of long COVID, a study employed an unsupervised machine learning method that utilized the semantic similarity of phenotype data to stratify patients with long COVID. This approach identified six clusters of PASC patients, which differed in terms of pre-existing comorbidities and the severity of acute COVID disease [13].

Our study differs from previous clustering studies in that it focused exclusively on patients with severe COVID-19, using a rigorous unsupervised machine learning approach with multiple techniques and internal validation metrics. The absence of distinct clusters in our homogeneous cohort suggests that the heterogeneity of PASC symptoms might be intrinsic rather than reflecting distinct phenotypes, at least in this specific population. This contribution is relevant for clinical practice as it highlights that standardized approaches to PASC treatment might not be appropriate for previously hospitalized patients with severe COVID-19, suggesting instead the need for highly personalized management strategies.

In our study, the application of a machine learning method in order to analyze the population of patients hospitalized for severe COVID-19 disease and who developed PASC confirmed the high heterogeneity of symptoms. However, this heterogeneity does not allow for the identification of common treatment pathways, confirming the need to create diagnosis and treatment pathways focused on every single patient.

To examine the influence of demographic factors on symptom patterns, we performed stratified clustering analyses by age group (≥65 vs. <65 years). These analyses did not reveal distinct or clinically meaningful clusters, consistent with findings from the overall cohort. Although we did not conduct regression-based adjustments for potential confounders, we acknowledge their value and propose this as an important direction for future research to better understand the relationship between patient characteristics and symptom profiles.

A possible limitation of our research is that symptoms were treated with equal weight, without considering frequency or severity. While this preserved specificity across patients, it may have limited the capacity to capture differences in symptom burden. However, by focusing on the presence or absence of symptoms at a standardized 3-month follow-up, we aimed to ensure comparability across patients and minimize recall bias, providing a consistent snapshot of PASC.

Another limitation might be that a complete-case approach was used to handle missing symptom data, leading to the exclusion of patients with more than 50% missing values. This decision was made to avoid introducing artifacts in the clustering process due to extensive imputation. However, only 19 patients (7.5%) were excluded, making it unlikely that this choice substantially affected the sample size or introduced significant selection bias. Nevertheless, future work will explore advanced imputation strategies, such as multiple imputation by chained equations (MICE), to further validate the stability of clustering results under different assumptions about missing data.

Finally, our study population was limited in size, and this aspect might hamper the identification of clearly separated clusters. However, the substantial homogeneity of the cohort, with all patients having been discharged after a severe COVID-19 infection, might justify the lack of distinct phenotypes.

The absence of clearly defined and clinically significant symptom clusters in our cohort has important implications for clinical practice. First, these findings strongly suggest that a “one-size-fits-all” approach to PASC management in this population might be inadequate. Instead, our results support the need for a highly personalized care strategy, where clinical assessment, therapeutic planning, and monitoring are tailored to each patient’s specific symptom manifestations and needs.

The observed heterogeneity might reflect the complexity of the pathophysiological mechanisms underlying PASC, which likely involve a combination of direct organ damage, persistent immune dysregulation, microvascular alterations, and psychosocial factors, all manifesting in varying proportions in individual patients. This hypothesis is supported by the high prevalence of multimorbidity (84.8%) in our cohort, which might further contribute to variability in symptom presentation and disease response.

From a practical perspective, our findings suggest that clinicians should adopt a holistic and patient-centered approach, systematically assessing the entire range of potential post-COVID symptoms and complications, rather than focusing on specific symptom clusters. Multidisciplinary teams, including specialists in internal medicine, pulmonology, cardiology, neurology, psychiatry, and rehabilitation, remain essential to adequately address these patients’ complex clinical pictures.

## 5. Conclusions

The extreme heterogeneity of patients affected by PASC has not allowed for the identification of specific symptom clusters even with the application of the most recent statistical techniques. The characteristics of the different cohorts of patients enrolled in previous studies may have been drivers for the emergence of cohort effects that make the results not generalizable.

In our study, enrolling a large cohort of consecutive patients with severe acute COVID-19 did not yield distinct or clinically meaningful symptom clusters at the 3-month follow-up. These findings underscore the complexity of post-COVID symptomatology and support the need for individualized diagnostic and therapeutic pathways rather than uniform protocols.

Based on our findings, several promising directions for future research emerge:

1. Integration of biomarkers: future phenotyping studies could benefit from the inclusion of inflammatory, immunological, metabolic, and specific organ damage biomarkers, which might reveal underlying patterns not evident from symptom analysis alone.
2. Multimodal imaging data: incorporating structural and functional imaging data could detect patterns of subclinical organ damage that might underlie reported symptoms.
3. Digital health data: the use of remote monitoring technologies could facilitate the longitudinal collection of physiological data in real-world settings, revealing temporal patterns not easily captured during standard clinical visits.
4. Integrated multi-omic approaches: high-resolution omic technologies could provide in-depth molecular characterization of PASC patients, potentially identifying distinctive molecular signatures.
5. Extended longitudinal analyses: longer-term follow-up studies (1–5 years) are needed to understand the natural history of PASC and identify predictors of symptom persistence or recovery.
6. Machine learning-based predictive models: developing models that integrate demographic, clinical, biological, and imaging data could help early identification of patients at risk for PASC.
7. Adaptive clinical trials: designing trials that dynamically respond to emerging data could accelerate the development of effective therapeutic strategies for PASC.

The implementation of these complementary research approaches could lead to a more nuanced understanding of the post-COVID syndrome, potentially revealing biologically distinct subtypes that require differentiated management strategies, even in the absence of clearly defined symptom clusters.

## Figures and Tables

**Table 1:** Subjects’ characteristics and disease main categories of the study population. Overall and by participating center. Disease categories were derived from the ICD9-CM discharge diagnosis codes and grouped according to the corresponding main chapter.

	ICD9-CM Chapter	Overall N = 382	San Matteo Hosp N = 242	Cremona Hosp N = 140
Sex (F)	-	102 (26.7%)	72 (29.8%)	30 (21.4%)
Age > 65	-	136 (35.7%)	84 (34.7%)	52 (37.4%)
Endotracheal intubation	-	97 (25.4%)	51 (21.1%)	46 (32.9%)
Multimorbidities	-	324 (84.8%)	200 (82.6%)	124 (88.6%)
Circulatory	7	176 (46.1%)	126 (52.1%)	50 (35.7%)
Endocrin	3	76 (19.9%)	66 (27.3%)	10 (7.1%)
Genitourinary	10	34 (8.9%)	24 (9.9%)	10 (7.1%)
Neurological	6	25 (6.5%)	21 (8.7%)	4 (2.9%)
Gastroenterological	9	13 (3.4%)	11 (4.5%)	2 (1.4%)
Cancer	2	12 (3.1%)	8 (3.3%)	4 (2.9%)
Hematological	4	10 (2.6%)	9 (3.7%)	1 (0.7%)
Dermatological	12	8 (2.1%)	5 (2.1%)	3 (2.1%)
Trauma	17	6 (1.6%)	4 (1.7%)	2 (1.4%)
Mental	5	5 (1.3%)	4 (1.7%)	1 (0.7%)
Musculoskeletal	13	4 (1.0%)	4 (1.7%)	0 (0.0%)
Other	18	157 (41.1%)	28 (11.6%)	129 (92.1%)
Symptoms	16	113 (29.6%)	7 (2.9%)	106 (75.7%)

**Table 2:** Prevalence of symptoms at follow-up (95%CI) overall and by participating center.

Symptom		All (N = 382)		San Matteo Hosp (N = 242)		Cremona Hosp (N = 140)
	N	% (95%CI)	N	% (95%CI)	N	% (95%CI)
Residual symptoms	253	67.8 (62.8, 72.5)	148	63.5 (56.9, 69.6)	105	75.0 (66.8, 81.8)
Multiple symptoms						
1	107	28.0 (23.6, 32.9)	71	29.3 (23.8, 35.6)	36	25.7 (18.9, 33.9)
2	77	20.2 (16.3, 24.6)	46	19.0 (14.4, 24.6)	31	22.1 (15.8, 30.1)
3+	74	19.4 (15.6, 23.8)	36	14.9 (10.8, 20.1)	38	27.1 (20.1, 35.4)
Dyspnea	170	60.9 (54.9, 66.6)	100	68.5 (60.2, 75.8)	70	52.6 (43.8, 61.3)
Fatigue	109	39.8 (34.0, 45.9)	64	45.7 (37.3, 54.3)	45	33.6 (25.8, 42.3)
Neuro-psychological symptoms	69	30.4 (24.6, 36.9)	33	35.9 (26.3, 46.6)	36	26.7 (19.6, 35.1)
Rheumatologic symptoms	47	21.1 (16.0, 27.1)	21	23.6 (15.5, 34.0)	26	19.4 (13.3, 27.3)
Cardiovascular symptoms	47	17.2 (13.0, 22.3)	28	20.3 (14.1, 28.2)	19	14.1 (8.9, 21.4)
Otorhinolaryngological symptoms	28	10.3 (7.1, 14.7)	20	14.5 (9.3, 21.7)	8	6.0% (2.8, 11.8)
Dermatologic symptoms	22	9.8 (6.4, 14.6)	6	6.7 (2.7, 14.5)	16	11.9 (7.1, 18.8)
Cough	18	6.6 (4.1, 10.5)	7	5.1% (2.3, 10.7)	11	8.1 (4.3, 14.4)
Gastrointestinal disorders	19	6.9 (4.3, 10.8)	16	11.4 (6.9, 18.2)	3	2.2 (0.6, 6.9)
Headache	11	4.9 (2.6, 8.9)	9	10.1 (5.0, 18.8)	2	1.5 (0.3, 5.8)

**Table 3:** Internal stability indexes for hierarchical (agglomerative and divisive), PAM, k-means, and DBSCAN clustering of patients.

Method	Average Silhouette	Separation Index (SI)	Cophenetic Correlation Coefficient	Entropy
Agglomerative Clustering	0.31	0.05	0.61	1.10
Divisive Clustering	0.31	0.03	0.74	0.74
PAM Clustering	0.18	0.01	-	1.27
K-Means	**0.19**	**0.26**	**-**	**0.69**
DBSCAN	**0.47**	**0.55**	**-**	**1.01**

In bold modals best performance.

## Data Availability

Data and codes available upon reasonable request.

## Supplementary Materials

The following supporting information can be downloaded at: https://www.mdpi.com/article/10.3390/jcm14113670/s1, Table S1: Set of variables recorded during outpatient visit at 3 months following the discharge. Table S2: Baseline characteristics across the three clusters identified by DBSCAN. Table S3: Baseline Characteristics of 19 patients excluded from the unsupervised machine learning approach vs 234 included. References from [35,36,37,38,39,40,41] mentioned in the Supplementary File.
