# Supplementary material for: Long COVID’s Hidden Complexity: Machine Learning Reveals Why Personalized Care Remains Essential
Source: J Clin Med. 2025 May 23;14(11):3670. doi: 10.3390/jcm14113670 (PMC12155299; doi:10.3390/jcm14113670)
Supplement: Supplementary file 1 [file jcm-14-03670-s001.zip › jcm-3644089-supplementary.pdf]

## 1) Unsupervised machine learning

Clustering is an unsupervised machine learning technique used to identify patterns or groups in data without any prior knowledge of them. Data points grouped within the same cluster are more similar to each other than to those in other clusters. This process helps uncover hidden structures in the data that may be complex to identify only by visual inspection.

In this study, clustering was applied to investigate the presence of patterns among patients with residual symptoms, collected at 3-months follow-up visit, correlated with post-acute COVID-19 syndrome (PACS). First, Principal Component Analysis (PCA), a statistical method used in multivariate data analysis for dimensionality reduction, was applied in order to explore the dataset and assess the clustering tendency. Also, non-linear dimensionality reduction techniques such as t-SNE and UMAP were applied and tested with a range of parameter settings. As shown in Supplementary Figure 1, none of these approaches revealed distinct or stable clusters in the symptom data.

The R package used for dimensionality reduction techniques were the following:

PCA: R Function *prcomp {stats v. 4.3.2}*

t-SNE: R Function *tsne {tsne v. 0.1-3.1}*

UMAP: R Function *umap {umap v. 0.2.10.0}*

Then, we applied the following clustering methods:

- Agglomerative Hierarchical Clustering (R Function*: hclust (method = "ward.D2") {stats v. 4.3.2}*), which begins by considering each data point as an individual cluster and gradually combines them in subsequent steps [1].
- Divisive Hierarchical Clustering (R Function*: diana {cluster v 2.1.6}*), starts by initially grouping all data into a single cluster and then iteratively dividing them into smaller clusters [2].
- Partition around medoids (PAM) (R Function: *pam {cluster v 2.1.6}*), a method that identifies cluster centres (medoids) and forms clusters around these central points, useful for robust clustering with non-standard data shapes [3].
- K-means (R Function: kmeans *{stats v. 4.3.2}),* a centroid-based method that partitions data into a predefined number of clusters. Hartigan and Wong algorithm was applied [4].
- DBSCAN clustering (R Function: dbscan *{dbscan v. 1.2.2}),* which identifies clusters based on density patterns, allowing for the detection of noise and non-linearly shaped clusters [5].

Given the mixed type of variables in the dataset (numeric and binary), Gower's method [6] was used to calculate a dissimilarity matrix. This step is an essential prerequisite for executing any clustering method, as it establishes a structured representation of the dissimilarities between data points.

All these clustering methods were performed along with parameter sensitivity analyses (e.g., varying the number of clusters for k-means and PAM, adjusting the eps value for DBSCAN). Even with these actions, all methods failed to identify reproducible or clinically meaningful subgroups.

We selected the "best performing" clustering method by considering the following validation metrics: cophenetic correlation coefficient (for hierarchical clustering) and average silhouette width. The cophenetic correlation coefficient assesses how faithfully a hierarchical clustering preserves the pairwise distances between data points, reflecting the similarity between the original distances and the hierarchical clustering. On the other hand, the average silhouette width measures the coherence of clusters by comparing the average distance between points within clusters against the average distance to points in the nearest neighbouring cluster, providing insight into cluster quality and separation. Additionally, two other measures of internal stability were computed:

- Separation Index [7], which measures the average distance between clusters, providing insight into distinct separation of clusters. A higher separation index signifies better-defined and more separated clusters, indicating stronger clustering performance.
- Entropy, which is a measure of uncertainty or disorder within clusters. A cluster with low entropy indicates that the majority of its data points belong to a single class or have similar characteristics, resulting in a more homogeneous and well-defined cluster. Conversely, a cluster with high entropy suggests that its data points are spread across multiple classes or have diverse characteristics, indicating a less coherent cluster.

All the analyses were performed with:

- Intel(R) Core(TM) i5-10400 CPU @ 2.90GHz, 8 GB RAM;
- R version 4.3.2 and the following additional packages: tidyverse (v 2.0.0), cluster (v 2.1.6), factoextra (v. 1.0.7), fossil (v 0.4.0), fpc (v 2.2-10).

**Supplementary Table 1:** Set of variables recorded during outpatient visit at 3 months following the discharge. The macro-categories are abbreviated as CV = CardioVascular, GI-GE = GastroIntestinal/Gastroesophageal, ORL = Otorhinolaryngological, RHE = Rheumatological).

| **Sex** | **GI_GE_reflux** | **Neuro_brain_fog** |
|---|---|---|
| Age at visit | GI_Abdominal_pain | Neuro_insomnia |
| Dyspnea | ORL | Neuro_memory_concentration_trouble |
| CV symptoms | ORL_Ageusia | Neuro_paresthesia_dysesthesia |

| | | |
|---|---|---|
| CV_chest_pain | ORL_Anosmia | Neuro_visual_impairment |
| CV_tachycardia | ORL_both | Neuro_other |
| CV_peripheral_edema | ORL_Earing_impairment | Rheumatologic symptoms |
| CV_other | Fatigue | RHE_arthralgia |
| Cough | Persistent Headache | RHE_myalgia |
| GI disorders | Neuro-psychological symptoms | RHE_other |
| GI_Nausea | Neuro_anxiety | Dermatologic symptoms |
| GI_Diarrhea | Neuro_depression | Derma_eruption |

2) **Supplementary Table 2: Baseline characteristics across the three clusters identified by DBSCAN.**

The table reports the distribution of baseline variables across the three patient clusters (Cluster 1: N = 87; Cluster 2: N = 47; Cluster 3: N = 32), with data presented as median (Q1, Q3) for continuous variables and n (%) for categorical variables. Statistical comparisons were performed using the Kruskal–Wallis rank-sum test for continuous variables and Pearson's chi-squared or Fisher's exact test for categorical variables, as appropriate. A false discovery rate (FDR) correction was applied to adjust for multiple testing.

Although some variables showed statistically significant differences between clusters (e.g., age, sex, fatigue, rheumatologic and dermatologic symptoms), the overall clinical interpretation of these clusters remains limited. No distinct or clinically meaningful phenotypes emerged from the clustering, suggesting that the identified groupings may not reflect biologically relevant subtypes within the cohort.

**Supplementary Table 2**: Patient characteristics of clusters identified by DBSCAN clustering.

| **Variable** | **1**<br>N = 87[1] | **2**<br>N = 47[1] | **3**<br>N = 32[1] | **p-value**[2] | **q-value**[3] |
|---|---|---|---|---|---|
| **Age** | 52.0 (46.0, 55.0) | 71.0 (66.0, 76.0) | 63.0 (60.0, 70.5) | <0.001 | <0.001 |
| **Sex (M)** | 71 (81.6%) | 23 (48.9%) | 5 (15.6%) | <0.001 | <0.001 |
| **Dyspnea** | 55 (63.2%) | 31 (66.0%) | 26 (81.3%) | 0.2 | 0.2 |
| **CV symptoms** | 22 (25.3%) | 5 (10.6%) | 3 (9.4%) | 0.040 | 0.072 |
| **Cough** | 6 (6.9%) | 2 (4.3%) | 3 (9.4%) | 0.6 | 0.6 |
| **GI disorders** | 7 (8.0%) | 0 (0.0%) | 4 (12.5%) | 0.042 | 0.072 |
| **ORL** | 8 (9.2%) | 1 (2.1%) | 5 (15.6%) | 0.085 | 0.11 |
| **Fatigue** | 54 (62.1%) | 47 (100.0%) | 3 (9.4%) | <0.001 | <0.001 |
| **Persistent Headache** | 9 (10.3%) | 1 (2.1%) | 0 (0.0%) | 0.072 | 0.11 |
| **Neuro-psychological symptoms** | 19 (21.8%) | 12 (25.5%) | 13 (40.6%) | 0.12 | 0.14 |
| **Rheumatologic symptoms** | 12 (13.8%) | 11 (23.4%) | 12 (37.5%) | 0.017 | 0.042 |
| **Dermatologic symptoms** | 4 (4.6%) | 3 (6.4%) | 7 (21.9%) | 0.015 | 0.042 |

[1] Median (Q1, Q3); n (%)

[2] Kruskal-Wallis rank sum test; Pearson's Chi-squared test; Fisher's exact test

[3] False discovery rate correction for multiple testing

3) **Supplementary Table 3: Baseline Characteristics of 19 patients excluded from the unsupervised machine learning approach vs 234 included.**

Comparison of key demographic and clinical characteristics between patients included in the clustering analysis and those excluded due to high symptom data missingness (>50%). This analysis was conducted to assess potential selection bias introduced by the exclusion criterion. No major differences were observed between included and excluded patients, except for gastrointestinal symptoms, which were more frequently reported among excluded individuals ($p < 0.001$). However, gastrointestinal symptoms were overall infrequent in the sample (n = 19), suggesting that this imbalance is unlikely to substantially impact the generalizability of the study findings.

**Supplementary Table 3. Patient characteristics of patient included vs. escluded from the analyses**

| **Variable** | **Excluded** N = 19[1] | **Included** N = 234[1] | **p-value**[2] |
|---|---|---|---|
| **Sex (M)** | 14 (73.7%) | 163 (69.7%) | 0.7 |
| **Dyspnea** | 12 (75.0%) | 159 (67.9%) | 0.6 |
| **CV symptoms** | 1 (11.1%) | 46 (19.7%) | >0.9 |
| **Cough** | 0 (0.0%) | 18 (7.7%) | >0.9 |
| **GI disorders** | 5 (45.5%) | 14 (6.0%) | <0.001 |
| **ORL** | 3 (37.5%) | 25 (10.7%) | 0.052 |
| **Fatigue** | 3 (30.0%) | 106 (45.3%) | 0.5 |
| **Persistent Headache** | 1 (5.3%) | 10 (4.3%) | 0.6 |
| **Neuro-psychological symptoms** | 6 (31.6%) | 58 (24.8%) | 0.6 |
| **Rheumatologic symptoms** | 3 (16.7%) | 43 (18.4%) | >0.9 |
| **Dermatologic symptoms** | 4 (21.1%) | 18 (7.7%) | 0.069 |

[1] n (%)

[2] Pearson’s Chi-squared test; Fisher’s exact test

**References**

1. Murtagh, F.; Legendre, P. Ward's hierarchical agglomerative clustering method: which algorithms implement Ward's criterion? *J. Classif.* **2014**, *31*, 274–295. https://doi.org/10.1007/s00357-014-9161-z

2. Kaufman, L.; Rousseeuw, P.J. *Finding Groups in Data: An Introduction to Cluster Analysis*; Wiley: John Wiley & Sons, Inc., Hoboken, NJ, USA 1990

3. Schubert, E.; Rousseeuw, P.J. (2019). Faster k-Medoids Clustering: Improving the PAM, CLARA, and CLARANS Algorithms. In *Similarity Search and Applications*, Proceedings of 15th International Conference, SISAP 2022, Bologna, Italy, October 5–7, 2022; Springer: New York City, NY, USA; pp. 171–187.

4. Hartigan, J.A.; Wong, M.A. Algorithm AS 136: A K-Means Clustering Algorithm. *J. R. Stat. Soc. Ser. C. Appl. Stat.* **1979**, *28,* 100–108.

5. Ester, M.; Kriegel, H.P.; Sander, J.; Xu, X. (1996). A density-based algorithm for discovering clusters in large spatial databases with noise. Proceedings of the Second International Conference on Knowledge Discovery and Data Mining (KDD-96), Portland, OR, USA, August 2–4 1996; AAAI Press: Washington, DC, USA; pp. 226–231.

6. Gower, J.C. A General Coefficient of Similarity and Some of Its Properties. *Biometrics*. **1971**, *27*, 857–871.

7. Hennig, C.; Liao, T.F. How to find an appropriate clustering for mixed-type variables with application to socio-economic stratification. *J. R. Stat. Soc. Ser. C. Appl.* **2013**, *62*, 309–369.
